# Managing psychosocial hazards in the workplace: how to link frequency and severity using risk matrices

**DOI:** 10.3389/fpsyg.2026.1753317

**Published:** 2026-04-17

**Authors:** Yacine Taibi, Andreas Müller, Silja Bellingrath, Ciel A. Neuhaus, Yannick A. Metzler

**Affiliations:** 1 Department of Work and Organizational Psychology, Institute of Psychology, University of Duisburg-Essen, Essen, Germany; 2 IfADo – Leibniz Research Centre for Working Environment and Human Factors, Dortmund, Germany

**Keywords:** job demands, occupational stress, psychosocial hazard, risk assessment, risk evaluation, work stress

## Abstract

**Introduction:**

Evaluating and prioritizing psychosocial hazards remains a challenge for both scientists and practitioners. In contrast to traditional chemical or physical risk assessment methods, psychosocial assessments generally do not record health‑related outcomes for prioritization or severity evaluation, but infer associations probabilistically at the population level. The risk-matrix approach (RMA) addresses these limitations by structuring prioritization around exposure levels and the magnitude of associated health-related impairment. However, the RMA has not yet been applied systematically to psychosocial risk assessment.

**Methods:**

We developed a risk-matrix–based prioritization framework using the Copenhagen Psychosocial Questionnaire (COPSOQ). Psychosocial hazards were represented on the COPSOQ’s exposure scale and compared across several exposure levels from low to very high. Harm was captured with three health-related outcomes ranging from proximal cognitive stress responses to more distal indicators of burnout and general health. Using data from a German steel company (*N* = 7,242), we estimated the magnitude of outcome differences across exposure levels for each hazard while accounting for key demographic and job-related characteristics. The risk matrix then translates these associations into expected outcome differences across exposure levels, alongside an indication of uncertainty.

**Results:**

Across outcomes, higher hazard exposure was associated with greater cognitive stress symptoms, higher burnout, and poorer general health. Associations were strongest for hazards such as detrimental environmental conditions, work–privacy conflict, and emotional demands, yielding substantial model-implied outcome differences at moderate to high exposure levels.

**Discussion:**

For subsequent risk-mitigation interventions, psychosocial hazards can be prioritised based on their model-implied health impact across exposure levels rather than single cut-offs. This work advances the RMA framework by integrating health-related outcomes directly into psychosocial risk prioritisation in applied settings and underscores.

## 1 Introduction

The detrimental effects of psychosocial work characteristics on employees’ mental and physical health have been consistently proven in numerous studies (Niedhammer et al., 2021; Kivimäki and Kawachi, 2015; Lang et al., 2012; Rau and Buyken, 2015; Taibi et al., 2021; Theorell et al., 2015). To maintain health and workability of employees, risk management of psychosocial work characteristics is essential. With the implementation of psychosocial risk assessment, work analysis and work design as key disciplines of industrial psychology have found their way into practice. The European Union has legally obliged employers to integrate psychosocial hazards into general risk assessment (EU Directive 89/391/EEC, 1989), and most EU countries have since established specific legislation to clarify employer responsibilities (Jain et al., 2022). Beyond Europe, however, legal obligations to consider psychosocial hazards in occupational safety remain rare (Chirico et al., 2019), and even within the EU, the degree of implementation varies considerably across countries (Beck and Lenhardt, 2019; Ertel et al., 2010; Eurofound and EU-OSHA, 2014).

Risk management is a systematic process to combat potential hazards at work and combines three elements: hazards, harm, and risk (Clarke and Cooper, 2004). A psychosocial hazard can be any psychosocial work characteristic potentially impairing employee health and well-being (Cox et al., 2002). Harm refers to adverse health consequences and their severity, spanning a wide range of outcomes such as mental health issues and cardiovascular disease(Boot et al., 2024). Risk is then described as the probability that a hazard will cause harm. The understanding of psychosocial hazards currently relies on an epidemiological thinking that links workplace hazards to health outcomes through population-level statistics (Semmer et al., 2005). In practice, this means that when we identify a psychosocial hazard, such as high workload or low job control, we can only presume its health impact based on statistical associations observed across larger studies (Pikhart and Pikhartova, 2015). We know these hazards relate to increased rates of, for example, mental health issues and reduced well-being, but we cannot assess at which level of severity and priority in a specific occupational setting. This indirect approach differs fundamentally from how we assess physical and chemical hazards in the workplace, where exceeding certain thresholds or exposure limits inevitably induces harm. This difference matters because risk assessment traditionally defines risk as the product of both probability (how likely) and severity (how harmful) of an adverse event (Clarke and Cooper, 2000; Ballard, 1992). Current psychosocial assessment focusses primarily on exposure frequency—how often workers encounter stressors assessed by questionnaires—without adequately incorporating the severity of health impacts in a specific occupational setting. This gap may lead to systematic underestimation of psychosocial risks and, consequently, inadequate prevention strategies and work design interventions (Taibi et al., 2022).

What further complicates this case is that data on psychosocial risk assessment most often stems from subjective questionnaire data which might be, in many cases, only available on a cross-sectional level, at least for the very first time an organization collects such data. Since cross-sectional self-report data is not suitable for determining causality (Bauhoff, 2014) practice and applied psychology face the difficulty of evaluating psychosocial hazards in a form that forbids drawing causal conclusions. How to reliably and validly evaluate the likelihood of a risk occurring from psychosocial hazards, and how such results can be translated into actionable information is still scarcely researched (Hudson, 2016; Gaskell et al., 2007; Metzler et al., 2019; Taibi et al., 2022). To date, industrial and organizational psychology has offered limited practice-oriented methods to support organizations in such cases. A key reason for this gap is the disconnect between how psychosocial risks are researched and how risk management operates in practice. Research establishes associations between hazards and health outcomes at the population level, but translating these associations into actionable organizational decisions requires (a) a format that is compatible with established risk management procedures, (b) a way to compare hazards with different exposure distributions, and (c) transparency about uncertainty. Currently, no standardized method bridges this gap.

There is an urgent need to develop methods addressing these methodological challenges in the process of psychosocial risk assessment for both practice and applied science (Schulte et al., 2024). Therefore, the aim of our study is to adapt the risk matrix approach (RMA) (Emblemsvag, 2012) used in traditional risk assessment to evaluate the risk of psychosocial hazards by mapping exposure frequency and harm severity. By doing so, we provide a standardized method to evaluate psychosocial hazards that is independent of the specific questionnaires or instruments used for hazard identification. This enables prioritizing risks according to their impact on employee health. First, we review established risk-evaluation procedures; next, we set out methodological steps to adapt the RMA for valid assessment of psychosocial hazards; finally, we demonstrate the approach with an empirical application using anonymized data from an organizational psychosocial risk assessment conducted in a manufacturing context.

### 1.1 Established approaches for risk evaluation

Psychosocial hazards emerge from the way work is designed, organized and managed and refer to almost every construct that has been researched in the scope of work stress in the past 50 years (Boot et al., 2024), like role conflicts, quantitative demands (e.g., workload, time pressure, and work pace), or job autonomy. The process of psychosocial risk assessment roughly comprises the steps of hazard identification, evaluation, and combating hazards. One major issue that still challenges practitioners and scientists is how to reliably evaluate and prioritize psychosocial hazards. This step in risk assessment is crucial since the following steps of deriving and implementing risk mitigating measures depend on a proper and sound risk evaluation to maintain and improve employee health and safety (Hudson, 2016; Gaskell et al., 2007; Metzler et al., 2019; Taibi et al., 2022). Although several measures and strategies exist for this purpose, corresponding approaches to evaluate which hazards require intervention differ substantially and are often tightly linked to the hazard identification tools used. For example, Oakman et al. (2022) show that tools range from observational ergonomic methods (e.g., RULA, REBA) to psychosocial survey instruments (e.g., COPSOQ, ERI, HSE Stress Indicator Tool). The majority of approaches to risk evaluation can be divided into three main types (Metzler et al., 2019; Taibi et al., 2022; Dettmers and Stempel, 2021): uniform cut-off procedures like measures of exposure-based frequency, descriptive reference figures like in job-exposure matrices, and cut-off value-based approaches equivalent to occupational exposure limits of chemical agents.

#### 1.1.1 Uniform cut-off procedures

The uniform cut-off procedure applies a single rule-of-thumb threshold across hazards; exceeding this threshold is interpreted as indicating at least a moderate level of exposure and thus a potential need for action (Metzler et al., 2019; Dettmers and Stempel, 2021; Taibi et al., 2022). Importantly, the choice of such thresholds is instrument- and context-dependent: some approaches rely on benchmark comparisons and color-coded performance bands rather than the scale midpoint (e.g., HSE sample report; Health and Safety Executive, 2022), while other instruments provide fixed interpretive bands for action planning (e.g., NOSACQ-50 interpretation guidance; National Research Centre for the Working Environment, 2026; see also Kines et al., 2011). These values are based on the logic that for a given work characteristic, a theoretically plausible and empirically proven linear relationship with certain health impairments exists (Theorell et al., 2015; Lang et al., 2012). It is then assumed that the expected health impact of a hazard is greater, the more employees report being exposed. A legitimate critique of this approach is the arbitrary definition of scale average threshold values indicating a need for action. This approach ignores the factual empirical hazard-harm relationship present in a specific sample or setting (Metzler et al., 2019).

#### 1.1.2 Reference figures

Reference figures allow organizations to benchmark their own ratings with available data of job-exposure matrices (e.g., database of the COPSOQ; Nübling et al., 2011). Based on instrument-specific rules for assessing deviations of own data from these reference figures, psychosocial hazards are then selected as risks, for example based on the extent of that deviation. However, this approach mostly lacks a theoretical or empirical rationale for assessing the most important hazards from the extent of a deviation from the reference figures (Rau, 2022). Furthermore, the use of reference figures can lead to wrong conclusions. If the reference figures as benchmarks already imply unfavorable working conditions, the comparison would not indicate any meaningful results.

Furthermore, the use of reference figures can lead to misleading conclusions in either direction. If benchmark values are derived from samples that are not representative or are systematically skewed (e.g., toward organizations that voluntarily participate and may already have comparatively strong health and safety management), comparisons may underestimate problems in other contexts; conversely, if reference figures already reflect generally unfavorable conditions, deviations from the benchmark may appear small despite meaningful local issues (Kines et al., 2011).

#### 1.1.3 Cut-off values

Third and last, in the cut-off value-based approach, empirical thresholds are calculated for example via ROC curves (receiver operating characteristic) in relation to an outcome of interest, aiming to differentiate individuals at risk versus those not at risk. Disadvantages of this approach are that cut-off values often relate to only one specific type of harm, for example depression. Previous studies have so far only considered a small number of possible outcomes (e.g., Zeike et al., 2018; Diebig and Angerer, 2021; Dettmers and Stempel, 2021). Consequently, some hazards may be unrelated to clinically diagnosed outcomes but still affect non-clinical outcomes such as job satisfaction.

### 1.2 The risk matrix approach as a unifying framework

**Figure 1:**
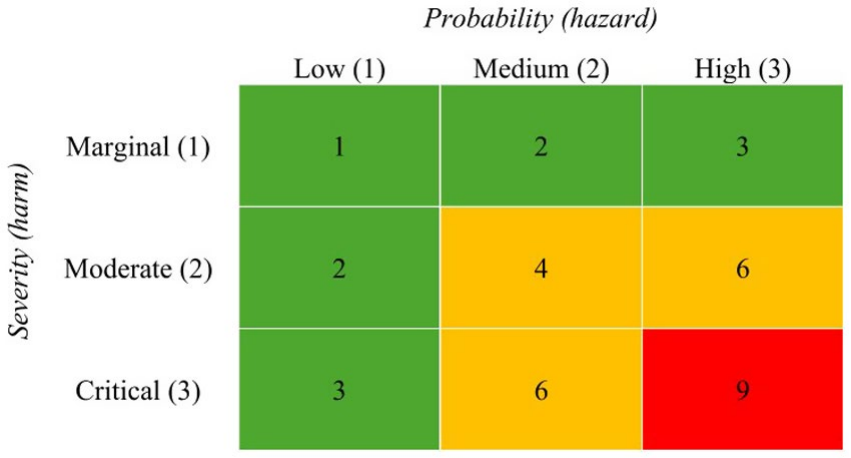
Exemplary illustration of a risk matrix. The numbers in cells quantify the risk for a harm by multiplying the two axes. The grid is divided into three different risk categories. Green indicates a low, yellow a medium and red a high risk. Source: Taibi et al. (2021); License: CC BY 4.0 adapted the material.

Risk assessment can be divided into qualitative, semi-quantitative and quantitative methods (Ni et al., 2010). While quantitative methods use numerical values to describe the extent of harm and/or frequency, qualitative methods present results as non-numerical estimates in the form of descriptions or recommendations. Assessing risks by a risk matrix is a semi-quantitative method (Levine, 2012). In risk matrices, risk is calculated as a combination of the probability or frequency of a hazard and its negative consequences, i.e., harm (Duijm, 2015). Both dimensions are scaled on a biaxial map (see Figure 1) with increasing levels of frequency and severity, respectively, (Ni et al., 2010). The map systematically displays different levels of probability or frequency supported by a color scheme and corresponding values to multiply the final risk index so that each cell of the map indicates the a certain need for action (Cox, 2008).

Although regularly criticized for being imprecise (Acebes et al., 2024), this critique originates predominantly from fields with access to diverse objective data sources – including sensor readings, historical incident records, and direct exposure measurements – that enable probabilistic, quantitative risk modeling (Cox, 2008; Duijm, 2015). In contrast, psychosocial risk assessment often relies on self-report questionnaire data which precludes such event-based probability estimation. This is precisely because the RMA can complement existing methods because it addresses some key challenges in psychosocial risk assessment that are not tackled by the above-described approaches: (a) consideration of potential differential risk effects of hazards, (b) consideration of different levels of harm, and (c) consideration of direct health effects within specific organizational settings. The RMA integrates health-related effects within the specific work context and thereby provides an organization-specific reference point for risk evaluation and prioritization. Risk is operationalized as a combination of (a) the likelihood of an adverse event estimated from organization-level risk assessment data and (b) the severity of its negative consequences, enabling an empirical, context-anchored linkage between psychosocial hazards and health-related effects (Taibi et al., 2022). Although such associations are typically interpreted by industrial and occupational experts, the RMA provides a credible starting point for a systematic and transparent evaluation. It can provide criterion-oriented thresholds for determining whether an exposure can be gradually classified as on a continuum from critical to non-critical related to health and well-being variables.

A central question is how such a matrix could be construed for evaluating psychosocial hazards—especially how to scale the axes of frequency and severity. Since psychosocial risk assessment is predominantly carried out by using questionnaires, the often Likert-scaled based ratings of a questionnaire already match the frequency axis. Most often, the Likert-scaling is constructed in such a way that respondents are asked to rate how frequently they experience the presence of a certain hazard (response options e.g.: always, often, sometimes, seldom, never / hardly). This corresponds to the frequency axis of a risk matrix that displays intervals of a continuous increase in frequency from low to high.

Conceptually, the severity of psychosocial harm can be indexed by the temporal latency of its manifestation and the degree to which impairments become global, persistent, and functionally integrated. Theoretical accounts of occupational stress consistently distinguish outcomes by how rapidly they emerge following exposure and how readily they resolve with recovery (Geurts and Sonnentag, 2006; Sonnentag, 2018). In psychosocial risk assessment, severity is thus not merely a matter of intensity but reflects a cumulative progression: from proximal, reversible strain reactions close to the source hazard, through intermediate states of chronic accumulation, to distal impairments that cut across multiple functional domains and are unlikely to resolve without intervention. This latency- and integration-based conception of severity provides a theoretically grounded basis for scaling the harm axis of the risk matrix (ISO 10075-1:2017, 2017).

To scale the axis of harm, it is necessary to reflect which theoretically sound and empirically validated outcomes could correspond to the levels of marginal, moderate and critical severity. There are several established theoretical models which consider the influence of unfavorable working conditions on health-related outcomes (e.g., job demands-control model, Karasek, 1979; job demands-resources model, Demerouti et al., 2001; effort-reward imbalance model, Siegrist, 2002; stress–strain concept, Rohmert, 1986). Outcomes vary from short to long-term effects like cardiovascular diseases (Kivimäki and Kawachi, 2015), musculoskeletal disease (Lang et al., 2012) depression or anxiety (Madsen et al., 2017). Classical conceptions of industrial psychology refer to classifying outcomes of psychosocial hazards into short, middle and long-term consequences (Semmer et al., 2005). In such classifications, the levels of severity are distinguished based on the time it takes for a negative effect to manifest, and the time needed for recovery from such an effect (Ford et al., 2014; Geurts and Sonnentag, 2006; Zapf et al., 1996). Considering short-term effects, fatigue, monotony, stress, or increased heart rate occur immediately and hence correspond to the marginal level of severity in the scope of the risk matrix. This level describes a reversable and less strong impact of a hazard. Middle and long-term effects refer to manifestations resulting from chronic stress, like low job satisfaction, personal burnout, or absenteeism, which can be grasped as moderate effects. Long-term consequences, corresponding to the critical level of severity, are especially cardiovascular incidents or diseases, clinical manifestations like depression, or psychosomatic diseases.

### 1.3 Study objectives

In adapting the RMA to psychosocial risk assessment, our aim is to go beyond a mere visualization of statistical coefficients. The framework fulfills four key functions. First, it bridges research evidence and occupational safety practice by embedding statistical associations within a format that is familiar to risk managers. Second, it provides a standardized and replicable procedure that can be applied independently of the specific questionnaire or instrument used for hazard identification. Third, it generates outputs that are accessible and meaningful to non-scientific stakeholders – such as employees, works councils, human resources departments, and occupational health services – who are responsible for making decisions based on the findings. Fourth, and most importantly, it enables differentiated prioritization: rather than classifying hazards simply as “critical” or “non-critical,” the matrix illustrates how expected health impacts vary across exposure levels, thereby supporting targeted interventions at specific exposure thresholds. This type of information is largely absent from existing approaches to psychosocial hazard evaluation, which typically operate without explicit reference to within-sample health-related data.

Using data from a large German steel manufacturing company (*N* = 7,242), we demonstrate how exposure–outcome associations can be translated into a standardized matrix format that supports evidence-informed prioritization decisions.

## 2 Materials and methods

### 2.1 Procedure and sample

The dataset derives from data collected during the psychosocial risk assessment in a large German steel manufacturing company. To assess psychosocial hazards, the company established a standardized process and concluded an agreement between the works committee and the management board to clarify and contract the procedure of psychosocial hazard analysis. Ethical considerations were approved by the ethical committee of the Leibniz-Research Center for Working Environment and Human Factors (ID 249).

All employees have access to rating their psychosocial work environment by means of the German version of the COPSOQ I (middle version Nübling et al., 2006). The questionnaire was completed in paper format during regular team and shift meetings near the workstations and was accompanied by trained personnel. Written informed consent was obtained from all voluntary participating employees. The current sample includes *N* = 7,242 employees. In addition, a variety of covariates were assessed. Gender, age in groups, type of work (blue or white collar), type of working contract (being fixed term- or permanent employed), years of experience on the current job, workload and working hours. The basic characteristics of the sample are shown in Table 1.

**Table 1:** Basic characteristics of the sample.

Variable	Percent %
Gender	
Male	88
Female	8
Missing	4
Age	
Up to 24 years	6
25–34 years	18
35–44 years	14
45–54 years	34
Over 55 years	22
Missing	5
Type of contract	
Fixed-term contract	10
Permanent contract	86
Missing	4
Working time	
Full-time	95
Part-time > 50%	2
Part-time < 50%	0.3
Missing	3
Type of work	
Blue collar	59
White collar	29
Missing	12
Work experience	
Under 1–5 years	29
6–10 years	15
11–20 years	16
21–30 years	16
More than 30 years	20
Missing	5
Working hours	
24 h service	1
Office Monday – Friday	28
Early shift	6
Full shift	50
Two shifts	7
Missing	8

*N* = 7,242.

### 2.2 Measures

The COPSOQ is not a new tool but rather consists of already existing scales for assessing work stress (i.e., psychosocial hazards) and respective health-related outcomes. The questionnaire contains the following dimensions for identifying psychosocial hazards: demands (14 items, subscales: quantitative demands, emotional demands, demands for hiding emotions, work-privacy conflict), influence and development (19 items, subscales: influence at work, degree of freedom, possibilities for development, meaning of work, workplace commitment), interpersonal relations and leadership (16 items, subscales: predictability, role-clarity, role-conflicts, quality of leadership, social support, feedback, social relations, sense of community, bullying), additional factors (18 items, subscales: trust and justice, job insecurity, environmental conditions; Metzler and Bellingrath, 2017). Outcomes are reflected by the subscales of intention to leave the job, job satisfaction, general health, personal burnout, cognitive stress symptoms, and satisfaction with life. All items use five-point Likert-type response options. Responses are recoded to a 0–100 scale (Always = 100, Often = 75, Sometimes = 50, Seldom = 25, Never/hardly ever = 0), resulting in a 0–100 range, with higher mean scores indicating greater exposure to hazards or higher experienced strain. Means, standard deviations and Cronbach’s *α* of all scales can be found in Table 2 in the appendix. Internal consistency was low for Social Relations (α = 0.45) and suboptimal for Presenteeism (α = 0.59). These scales contain only a small number of items, which can constrain Cronbach’s alpha; additionally, the values may reflect sample- or context-specific measurement characteristics. Accordingly, results involving these scales are interpreted cautiously because measurement error can attenuate associations. However, we decided to keep these variables in line with the study objectives to reflect real-world application scenarios where organizations may work with standardized instruments that include scales with variable psychometric properties. This illustrates how the matrix approach handles imperfect measurement situations common in applied settings.

**Table 2:** Means, standard deviations and Cronbach’s α of COPSOQ scales.

Scale	Mean (SD)	Ref.	Cronbach’s α	*N* of items
Quantitative demands	48 (18)	55	0.73	4
Emotional demands	44 (21)	52	0.81	3
Demands for hiding emotions	36 (25)	46	0.76	2
Work privacy conflict	37 (26)	42	0.91	5
Influence at work	59 (21)	58	0.76	4
Degrees of freedom	52 (21)	47	0.72	4
Possibilities for development	40 (20)	33	0.78	4
Meaning of work	30 (20)	26	0.81	3
Commitment	46 (20)	43	0.74	4
Predictability	50 (21)	46	0.72	2
Role clarity	30 (17)	27	0.82	4
Role conflicts	48 (20)	44	0.77	4
Quality of leadership	44 (22)	50	0.89	4
Social support	33 (20)	36	0.81	4
Feedback	52 (23)	58	0.68	2
Social relations	42 (22)	48	0.45	2
Sense of community	25 (19)	25	0.85	3
Signs of bullying	28 (26)	21	–	1
Trust and justice	43 (17)	–	0.78	4
Job insecurity	43 (25)	32	0.77	4
Environmental conditions	44 (18)	16	0.85	10
Intention to leave the job	16 (24)	16	–	1
Job satisfaction	37 (15)	37	0.82	7
General health	24 (21)	29	–	1
Absenteeism	29 (24)	–	–	1
Personal burnout	43 (19)	42	0.90	6
Presenteeism	32 (18)	–	0.59	3
Cognitive stress symptoms	28 (20)	29	0.88	4
Satisfaction with life	33 (17)	34	0.87	5

*N* = 7,242; Ref. = Reference values (Nübling et al., 2011).

### 2.3 Statistical analyses

R Studio (2023.06.0 Build 421) was used for all analyses. Missing data were handled using multiple imputation. Overall, 33% of participants had at least one missing value, while the proportion of missing values across all cells was 4.0%. We examined missingness descriptively and via Little’s MCAR test, which was statistically significant, *χ*^2^ (7061) = 10030.9, *p* < 0.001, suggesting the data were unlikely to be missing completely at random (Little, 1988). Given the sensitivity of Little’s test in large samples and observed associations between missingness and work characteristics (e.g., occupational group/type of work), we treated MCAR as implausible and adopted MAR as a working assumption conditional on observed variables, while acknowledging that MNAR cannot be ruled out (Dong and Peng, 2013; Enders, 2010).

We conducted multiple imputation by chained equations using mice package (*m* = 40; 20 iterations), including all psychosocial hazard scales, all health-related outcomes, and all covariates (gender, age, type of work, working hours, work experience, type of contract, workload); continuous variables were imputed using predictive mean matching, and categorical variables using appropriate regression models (White et al., 2011; van Buuren and Groothuis-Oudshoorn, 2011; Rubin, 1987). Models were estimated in each imputed dataset and pooled using Rubin’s rules to obtain pooled coefficients, standard errors, and 95% confidence intervals (Rubin, 1987). The variable “type of work” showed comparatively higher item nonresponse. In organizational surveys, such missingness may reflect reluctance to disclose job classification information (e.g., privacy or identifiability concerns). We addressed this by treating missingness within the multiple imputation framework and by adjusting substantive models for type of work as a covariate.

### 2.4 Constructing the matrix and calculating the risk score

In the following section, we describe the steps for constructing a risk matrix as a decision-support tool for occupational psychosocial risk assessment. Importantly, COPSOQ hazard ratings reflect experienced exposure frequency (i.e., how often a respondent is exposed to a psychosocial condition). Therefore, the exposure axis of the matrix can be expressed directly on the COPSOQ 0–100 scale. All hazard scales were coded such that higher values indicate more adverse exposure.

We operationalized harm using three COPSOQ health-related outcomes that represent increasingly distal indicators of impairment: cognitive stress symptoms (proximal strain), personal burnout (intermediate strain), and general health (distal cross-domain strain). In COPSOQ, cognitive stress items are explicitly framed with a recent reference period (e.g., “during the past 4 weeks”), supporting their use as indicators of current/near-term strain and exhaustion. Personal burnout was measured with the Copenhagen Burnout Inventory personal burnout scale (Kristensen et al., 2005). General health was assessed using the EQ-5D visual analog scale (EQ VAS), a 0–100 self-rated health measure reflecting respondents’ overall health judgment (EuroQol Group, 1990, 2025; Herdman et al., 2011; Scalone et al., 2013; Janssen et al., 2013). We use these outcomes as continuous self-report indicators to represent a spectrum of health-related impairment ordered by the latency of their manifestation: from proximal, rapidly emerging strain responses (cognitive stress symptoms), through more sustained exhaustion requiring prolonged exposure to develop (personal burnout), to a distal indicator that reflects cumulative health deterioration over time (EQ VAS).

To quantify the empirical association between psychosocial hazards and each outcome while preserving measurement information, we estimated one multivariable linear regression model per outcome, with the respective outcome as dependent variable and all psychosocial hazards entered simultaneously as predictors, adjusting for gender, age, type of work, working hours, work experience, type of contract, and workload. The pooled unstandardized regression coefficient (B) represents the expected change in the outcome (in scale points) associated with a one-point increase in the focal hazard score, conditional on the remaining hazards and covariates. Table 3 reports pooled B, standard errors, and 95% confidence intervals for each hazard–outcome association. To increase model transparency, pooled R^2^ and adjusted R^2^ were summarized, and model assumptions were assessed using standard residual diagnostics together with Breusch–Pagan tests for heteroscedasticity and RESET tests as indicators of possible functional-form misspecification (see Supplementary Table S1).

**Table 3:** Pooled linear regression coefficients for psychosocial hazards predicting continuous health outcomes (multiple imputation).

Outcome	Predictor	B	SE	*t*	*p*	95% CI
Cognitive stress symptoms	Quantitative demands	−0.021	0.015	−1.392	0.164	[−0.050, 0.009]
	Emotional demands	0.083	0.013	6.587	<0.001	[0.058, 0.108]
	Demands for hiding emotions	0.029	0.010	2.773	0.006	[0.008, 0.049]
	Work-privacy conflict	0.134	0.010	13.466	<0.001	[0.114, 0.153]
	Limited influence at work	0.023	0.012	1.964	0.050	[0.000, 0.045]
	Restricted degrees of freedom	−0.043	0.013	−3.275	0.001	[−0.069, −0.017]
	Limited opportunities for development	0.020	0.014	1.448	0.148	[−0.007, 0.048]
	Reduced meaning of work	0.051	0.015	3.458	<0.001	[0.022, 0.080]
	Reduced commitment	−0.021	0.013	−1.569	0.117	[−0.047, 0.005]
	Limited predictability	−0.036	0.012	−2.901	0.004	[−0.061, −0.012]
	Low role clarity	0.174	0.015	11.690	<0.001	[0.144, 0.203]
	Role conflicts	0.063	0.012	5.046	<0.001	[0.039, 0.088]
	Poor leadership quality	−0.011	0.013	−0.842	0.400	[−0.036, 0.014]
	Insufficient social support	0.000	0.016	0.028	0.977	[−0.030, 0.031]
	Insufficient feedback	−0.014	0.011	−1.243	0.214	[−0.036, 0.008]
	Poor social relations	−0.008	0.010	−0.861	0.389	[−0.027, 0.011]
	Weak sense of community	0.103	0.014	7.553	<0.001	[0.077, 0.130]
	Signs of bullying	0.071	0.009	7.861	<0.001	[0.053, 0.089]
	Low trust and justice	0.019	0.015	1.220	0.223	[−0.011, 0.049]
	Job insecurity	0.087	0.009	9.813	<0.001	[0.070, 0.104]
	Detrimental environmental conditions	0.155	0.014	10.816	<0.001	[0.127, 0.183]
Personal burnout	Quantitative demands	−0.002	0.013	−0.157	0.875	[−0.028, 0.024]
	Emotional demands	0.174	0.011	15.531	<0.001	[0.152, 0.196]
	Demands for hiding emotions	0.018	0.009	1.933	0.053	[−0.000, 0.036]
	Work-privacy conflict	0.202	0.009	23.212	<0.001	[0.185, 0.219]
	Limited influence at work	0.043	0.010	4.301	<0.001	[0.023, 0.063]
	Restricted degrees of freedom	−0.011	0.012	−0.922	0.356	[−0.034, 0.012]
	Limited opportunities for development	0.055	0.012	4.485	<0.001	[0.031, 0.079]
	Reduced meaning of work	0.051	0.013	4.041	<0.001	[0.026, 0.076]
	Reduced commitment	0.022	0.012	1.874	0.061	[−0.001, 0.045]
	Limited predictability	−0.021	0.011	−1.922	0.055	[−0.042, 0.000]
	Low role clarity	0.052	0.013	4.003	<0.001	[0.027, 0.078]
	Role conflicts	0.060	0.011	5.442	<0.001	[0.038, 0.081]
	Poor leadership quality	0.014	0.011	1.280	0.201	[−0.008, 0.036]
	Insufficient social support	0.001	0.014	0.047	0.963	[−0.026, 0.027]
	Insufficient feedback	−0.008	0.010	−0.771	0.441	[−0.027, 0.012]
	Poor social relations	−0.010	0.008	−1.152	0.250	[−0.026, 0.007]
	Weak sense of community	0.061	0.012	5.160	<0.001	[0.038, 0.085]
	Signs of bullying	0.051	0.008	6.373	<0.001	[0.035, 0.067]
	Low trust and justice	0.036	0.014	2.614	0.009	[0.009, 0.062]
	Job insecurity	0.102	0.008	13.217	<0.001	[0.087, 0.117]
	Detrimental environmental conditions	0.180	0.012	14.416	<0.001	[0.155, 0.204]
General health	Quantitative demands	−0.086	0.019	−4.575	<0.001	[−0.123, −0.049]
	Emotional demands	0.016	0.016	0.981	0.327	[−0.016, 0.047]
	Demands for hiding emotions	0.032	0.013	2.377	0.018	[0.006, 0.058]
	Work-privacy conflict	0.086	0.013	6.778	< 0.001	[0.061, 0.111]
	Limited influence at work	−0.008	0.014	−0.545	0.585	[−0.036, 0.020]
	Restricted degrees of freedom	0.039	0.017	2.252	0.024	[0.005, 0.072]
	Limited opportunities for development	−0.004	0.018	−0.221	0.825	[−0.039, 0.031]
	Reduced meaning of work	0.000	0.019	0.013	0.989	[−0.036, 0.037]
	Reduced commitment	0.045	0.017	2.739	0.006	[0.013, 0.078]
	Limited predictability	−0.036	0.015	−2.334	0.020	[−0.066, −0.006]
	Low role clarity	0.059	0.019	3.173	0.002	[0.022, 0.095]
	Role conflicts	0.025	0.015	1.615	0.107	[−0.005, 0.055]
	Poor leadership quality	0.046	0.016	2.893	0.004	[0.015, 0.078]
	Insufficient social support	0.020	0.020	1.028	0.304	[−0.018, 0.058]
	Insufficient feedback	−0.011	0.015	−0.734	0.463	[−0.039, 0.018]
	Poor social relations	−0.010	0.012	−0.833	0.405	[−0.034, 0.014]
	Weak sense of community	0.076	0.017	4.394	<0.001	[0.042, 0.110]
	Signs of bullying	0.041	0.012	3.455	<0.001	[0.018, 0.065]
	Low trust and justice	−0.003	0.020	−0.162	0.871	[−0.042, 0.036]
	Job insecurity	0.000	0.011	0.025	0.980	[−0.022, 0.022]
	Detrimental environmental conditions	0.041	0.018	2.331	0.020	[0.007, 0.076]

B = unstandardized pooled regression coefficient; SE = pooled standard error; CI = 95% confidence interval. Coefficients are pooled across 40 multiply imputed datasets (MICE; predictive mean matching for continuous scales). All models were adjusted for gender, age, type of work (blue- vs. white-collar), workload, working hours, work experience, and type of contract. All psychosocial hazard predictors were coded such that higher values indicate more adverse conditions. Resource-oriented scales were therefore relabelled in the adverse direction to facilitate interpretation. Outcome labels were also aligned so that higher values consistently indicate greater impairment or less favorable functioning. Positive coefficients therefore indicate that more adverse exposure is associated with poorer outcomes.

For the risk matrix, we translate each pooled coefficient into model-implied outcome changes at fixed exposure points (25/50/75/100) relative to 0 (“never/hardly ever”). Each matrix cell therefore represents the expected change in the outcome at exposure level x compared with 0, alongside its 95% confidence interval, and should be interpreted as an association-based estimate conditional on the remaining hazards and covariates. Because the matrix is intended for transparent communication and prioritization in applied settings, we display the resulting matrix values separately for each hazard, although all estimates are derived from the same outcome-specific multivariable models.

## 3 Results

We next report the results from the multiple-imputation (*m* = 40) pooled linear regression models using continuous health outcomes. For each outcome, we estimated one covariate-adjusted multivariable model including all psychosocial hazards simultaneously (adjusted for gender, age, type of work, working hours, work experience, type of contract, and workload). Table 3 summarizes the pooled unstandardized coefficients (B), standard errors, and 95% confidence intervals. The coefficient B represents the expected change in the outcome score (0–100) associated with a one-point increase in the focal hazard score (0–100), conditional on the remaining hazards and the covariates.

Across hazards, higher exposure was generally associated with higher levels of cognitive stress symptoms and exhaustion and with poorer subjective general health (coded such that higher values indicate greater impairment), although the magnitude of these adjusted associations varied considerably once shared variance among hazards was taken into account. Given the large sample size, interpretation focuses on the magnitude and consistency of model-implied outcome differences rather than statistical significance alone. In the multivariable models, comparatively strong positive associations were observed in particular for work–privacy conflict, emotional demands, job insecurity, and detrimental environmental conditions, whereas several other coefficients were attenuated and some no longer showed confidence intervals excluding zero after simultaneous adjustment for the remaining hazards (see Table 3). For example, a 1-point increase in work–privacy conflict was associated with increases of 0.134 points in cognitive stress symptoms and 0.202 points in exhaustion, while a 1-point increase in detrimental environmental conditions was associated with increases of 0.155 points in cognitive stress symptoms and 0.180 points in exhaustion. Associations with impaired subjective general health were generally smaller in magnitude, although work–privacy conflict and detrimental environmental conditions also showed positive adjusted associations for this outcome.

To facilitate interpretation in the risk matrix, we translated each pooled coefficient into model-implied outcome changes (Δŷ) at fixed exposure levels (25/50/75/100) relative to the reference level of 0 (“never/hardly ever”), while holding the remaining hazards and covariates constant. The resulting matrices present these adjusted Δŷ values and their 95% confidence intervals for each hazard across the three outcomes (for examples, see Tables 4, 5; all matrices are provided in Supplementary Table S2). A hazard level of 75 on the work–privacy conflict scale corresponded to expected increases of Δŷ = 10.02 points in cognitive stress symptoms, Δŷ = 15.14 points in exhaustion, and Δŷ = 6.45 points in impaired subjective general health relative to zero exposure. Likewise, a hazard level of 75 on the detrimental environmental conditions scale corresponded to Δŷ = 11.64 points in cognitive stress symptoms, Δŷ = 13.46 points in exhaustion, and Δŷ = 3.09 points in impaired subjective general health. We present all hazards and exposure levels without post-hoc filtering and interpret estimates together with their confidence intervals to reflect uncertainty.

**Table 4:** Risk-matrix values for emotional demands.

Hazard level	Cognitive stress symptoms	Personal burnout	General health
0	0.00 [0.00, 0.00]	0.00 [0.00, 0.00]	0.00 [−0.00, 0.00]
25	4.35 [3.80, 4.89]	2.08 [1.46, 2.70]	0.40 [−0.39, 1.19]
50	8.69 [7.59, 9.79]	4.16 [2.92, 5.39]	0.79 [−0.79, 2.37]
75	13.04 [11.39, 14.68]	6.23 [4.38, 8.09]	1.19 [−1.18, 3.56]
100	17.38 [15.19, 19.58]	8.31 [5.84, 10.78]	1.58 [−1.58, 4.74]

Hazard-specific adjusted matrix values derived from outcome-specific multivariable pooled linear regression models estimated across the multiply imputed datasets (MICE). For each outcome, all psychosocial hazards were entered simultaneously, together with the covariates gender, age, type of work, working hours, work experience, type of contract, and workload. Cells show the predicted change in outcome score (0–100) relative to the reference level (hazard = 0; “never/hardly ever”) at hazard levels 0/25/50/75/100, while holding the remaining hazards and covariates constant; values in brackets are 95% confidence intervals. Higher values indicate worse health for all outcomes.

**Table 5:** Risk-matrix values for work-privacy conflict.

Hazard level	Cognitive stress symptoms	Personal burnout	General health
0	0.00 [0.00, 0.00]	0.00 [0.00, 0.00]	0.00 [0.00, 0.00]
25	5.05 [4.62, 5.47]	3.34 [2.86, 3.83]	2.15 [1.53, 2.77]
50	10.10 [9.24, 10.95]	6.68 [5.71, 7.66]	4.30 [3.06, 5.55]
75	15.14 [13.87, 16.42]	10.02 [8.57, 11.48]	6.45 [4.58, 8.32]
100	20.19 [18.49, 21.90]	13.37 [11.42, 15.31]	8.60 [6.11, 11.09]

Hazard-specific adjusted matrix values derived from outcome-specific multivariable pooled linear regression models estimated across the multiply imputed datasets (MICE). For each outcome, all psychosocial hazards were entered simultaneously, together with the covariates gender, age, type of work, working hours, work experience, type of contract, and workload. Cells show the predicted change in outcome score (0–100) relative to the reference level (hazard = 0; “never/hardly ever”) at hazard levels 0/25/50/75/100, while holding the remaining hazards and covariates constant; values in brackets are 95% confidence intervals. Higher values indicate worse health for all outcomes.

Model-fit summarizes for the outcome-level multivariable models are reported in Supplementary Table S1. Pooled adjusted R^2^ values were 0.289 for cognitive stress symptoms, 0.450 for personal burnout, and 0.100 for general health, with only minimal variation across imputations. Diagnostic checks indicated heteroscedasticity across all three models and mixed RESET results, with no indication of misspecification for personal burnout, inconsistent evidence for cognitive stress symptoms, and more consistent indications for general health. These findings suggest that the linear specification provides a useful approximation for the present matrix framework, while associations involving general health should be interpreted with greater caution.

## 4 Discussion

Our study suggests that adapting the Risk Matrix Approach (RMA) to psychosocial risk assessment can offer practical advantages over commonly used evaluation approaches. Rather than relying solely on population-based evidence or external thresholds, the proposed matrix provides an organizationally embedded, model-based summary of how psychosocial hazard exposure relates to multiple health-related outcomes within the studied workplace context. This format supports prioritization decisions by making the expected outcome differences across exposure levels explicit and comparable across hazards and outcomes, while also communicating uncertainty via confidence intervals.

To clarify the nature of our contribution: The statistical methods underlying the risk matrix – linear regression with covariate adjustment – are standard. The contribution lies not in novel analytics but in the systematic translation of exposure-outcome associations into a decision-support framework for occupational practice. The matrix format partly aligns with how physical and chemical risks are already managed in organizations, thereby lowering the barrier for integrating psychosocial hazards into existing risk management systems. By providing regression-based estimates with confidence intervals across exposure levels, the framework allows practitioners to prioritize hazards based on expected health impact while remaining transparent about statistical uncertainty.

While the RMA is conceptually rooted in causal risk thinking, the present study should be interpreted as an associational application that quantifies empirical links between psychosocial hazard exposure and health-related outcomes in a specific organizational context. Accordingly, the matrix expresses model-implied differences in continuous outcomes across exposure levels and does not estimate event probabilities or causal effects. Because the data is cross-sectional and based on self-reports, the observed patterns cannot establish temporal ordering or exclude alternative explanations. Future longitudinal and intervention studies are required to test the causal pathways implicitly assumed by the matrix and to evaluate whether changes in psychosocial hazards lead to subsequent improvements in health-related outcomes. A related aspect is the level of analysis. Psychosocial risk assessment data are typically collected within organizational units—like teams, departments, or shifts—yet most approaches, including the present implementation, follow a single-stressor approach (Metzler et al., 2019) without explicitly modeling this nested structure. This omission may underestimate standard errors when observations within units are correlated. More fundamentally, it reflects a broader gap in current practice: psychosocial hazards are usually examined in isolation, even though employees may experience them as configurations within shared work environments. Future research could address both issues by applying multilevel modeling frameworks that simultaneously account for organizational clustering and allow hazard effects to vary across contexts. Such extensions would move the RMA closer to representing the joint, context-dependent nature of psychosocial risk.

Theoretical comparisons with other risk evaluation methods (e.g., uniform cut-off procedures and reference value-based approaches) indicate that the matrix provides a more differentiated basis for risk prioritization. Reference value-based approaches typically flag hazards when organizational mean values deviate from external norms (e.g., COPSOQ reference values; Nübling et al., 2011), yielding a binary signal: the hazard either exceeds the threshold or it does not. In contrast, our matrix links hazards to outcomes across multiple exposure levels by reporting model-implied outcome changes, providing decision-relevant information along a continuum rather than a single binary classification. Illustratively, for work–privacy conflict, the pooled coefficient for personal burnout was B = 0.202 (Table 3), implying an expected difference of 6.68 points at an exposure level of 50 and 10.02 points at an exposure level of 75 compared with 0, conditional on covariates. This gradual information allows organizations to address multiple decision points: at which exposure threshold does intervention become warranted? How should hazards be prioritized when resources are limited? Should intervention intensity be calibrated differently at moderate versus high exposure? The categorization into low/moderate/high exposure (≥50, ≥75 points) offers orientation points for such decisions, but the underlying continuous exposure–outcome relationship enables more flexible risk evaluation than uniform cut-off procedures that reduce hazard information to a single binary signal.

Across hazards, the direction of associations was largely consistent with the broader literature on psychosocial work characteristics and employees’ mental and physical health (Leineweber et al., 2019; Niedhammer et al., 2021; Taouk et al., 2020). As a result, the matrix communicates how expected outcome differences increase across exposure points under the assumed linear specification. For transparency and interpretability, hazards were modeled linearly on the 0–100 scale; future research could examine potential non-linear exposure–outcome relationships. This is particularly relevant for hypotheses about non-monotonic relationships (e.g., inverted U-shapes) that have been discussed for certain constructs in relation to performance outcomes (Bruggen, 2015; Pindek et al., 2023). While much of the evidence on such patterns concerns performance rather than health, testing non-linearity is an important next step for extending the present framework.

The RMA traditionally serves two purposes: determining risk acceptability and prioritizing intervention targets. In conventional applications, traffic light systems visually communicate risk levels; green indicates acceptable risk, yellow suggests monitoring or possible action, and red signals unacceptable risk requiring immediate intervention (Duijm, 2015). These thresholds can be adjusted based on organizational risk tolerance (Markowski and Mannan, 2008) or prevention philosophies, with some organizations setting stricter limits to emphasize early intervention. However, these acceptability thresholds remain largely subjective, depending on expert judgment rather than empirical evidence (Cox, 2008). While risk acceptability assessment is theoretically possible with the RMA, we deliberately focus on its second application: prioritization of hazards for intervention. This decision reflects significant limitations in applying acceptability thresholds to psychosocial hazards. The subjective nature of color-zone boundaries becomes particularly problematic for psychosocial risks, where health impacts vary considerably between individuals. Additionally, the “risk ties” problem, where different hazards receive identical scores despite posing qualitatively different risks, can lead to false equivalencies in risk acceptance decisions (Ni et al., 2010). Therefore, our framework uses the RMA primarily as a ranking tool to systematically prioritize psychosocial hazards based on the magnitude of model-implied health impact across exposure levels (Cox, 2008). As a result, we attain information that even comparatively infrequent exposures may warrant priority attention when they are associated with relatively larger outcome changes (Taibi et al., 2022). This approach shifts focus from asking “what risks can we accept?” to “which risks should we address first?”—a more pragmatic question for workplace interventions.

### 4.1 Practical application: from regression to prioritization

To facilitate replication and practical use, we illustrate the step-by-step process of constructing and interpreting the risk matrix using a worked example.

#### 4.1.1 Step 1: Specify the regression model

For each health outcome, we estimate a multivariable linear regression model. All psychosocial hazards are entered simultaneously as predictors, and the model adjusts for available covariates. The coefficient βⱼ for a given hazard represents the expected change in the outcome (on the 0–100 scale) associated with a one-point increase in that hazard, holding all other hazards and covariates constant.

#### 4.1.2 Step 2: Obtain the regression coefficient

Consider emotional demands predicting personal burnout. The pooled regression coefficient from the multiply imputed data was B = 0.174 (SE = 0.011, 95% CI [0.152, 0.196]; see Table 3). This indicates that each 1-point increase on the emotional demands scale is associated with a 0.174-point increase in personal burnout, conditional on the remaining hazards and covariates.

#### 4.1.3 Step 3: Transform the coefficient into matrix values

The matrix displays expected outcome differences at fixed exposure levels (0, 25, 50, 75, 100) relative to the reference level of 0 (“never/hardly ever”). Because the model is linear, these values are obtained by multiplying the coefficient by the exposure level. The 95% confidence intervals are derived by applying the same transformation to the lower and upper bounds of the coefficient’s CI:

- Exposure = 0 (reference): Δŷ = 0.00 [0.00, 0.00].
- Exposure = 25 (“seldom”): Δŷ = 0.174 × 25 = 4.35 [0.152 × 25, 0.196 × 25] = 4.35 [3.80, 4.90].
- Exposure = 50 (“sometimes”): Δŷ = 0.174 × 50 = 8.70 [7.60, 9.80].
- Exposure = 75 (“often”): Δŷ = 0.174 × 75 = 13.05 [11.40, 14.70].
- Exposure = 100 (“always”): Δŷ = 0.174 × 100 = 17.40 [15.20, 19.60].

These values populate the corresponding cells of the risk matrix (see Table 4).

#### 4.1.4 Step 4: Interpret the matrix for a single hazard

The matrix shows that employees reporting emotional demands at level 75 (“often”) are expected to score approximately 13 points higher on personal burnout than employees reporting no such demands, while holding the remaining hazards and covariates constant. The confidence interval [11.40, 14.70] indicates relatively high precision for this estimate.

#### 4.1.5 Step 5: Compare hazards for prioritization

The practical value of the matrix lies in comparing expected health impacts across hazards at the same exposure level. For instance, at exposure level 75, the model-implied increases in personal burnout are:

- Work–privacy conflict: Δŷ = 15.14 [13.87, 16.42].
- Emotional demands: Δŷ = 13.05 [11.40, 14.70].
- Detrimental environmental conditions: Δŷ = 13.46 [11.63, 15.30].

This comparison indicates that, within the present sample and after simultaneous adjustment for the remaining hazards, work–privacy conflict shows the strongest association with personal burnout at this exposure level, although the estimates remain subject to overlapping uncertainty. It is followed by environmental conditions and emotional demands. Such comparisons can help practitioners identify which hazards warrant priority attention when resources for intervention are limited.

#### 4.1.6 Step 6: Derive intervention priorities

In practice, prioritization should combine two sources of information: (a) the strength of the model-implied association with health outcomes and (b) the observed level or prevalence of exposure in the organization. A hazard is therefore likely to warrant priority attention when employees report elevated exposure and the matrix indicates substantial expected differences in health outcomes. For example, if work–privacy conflict or detrimental environmental conditions are reported at comparatively high levels in a given organizational unit, the matrix suggests that these hazards may be associated with meaningful increases in personal burnout and may therefore be suitable starting points for targeted interventions. By contrast, hazards with comparatively small coefficients or wide confidence intervals including zero may be assigned lower priority, even when exposure is not negligible.

The procedure described here can be applied to any dataset containing hazard and outcome measures on comparable scales. By generating organization-specific matrices, practitioners can move beyond generic benchmarks toward evidence-informed prioritization tailored to their own workforce.

### 4.2 Study limitations

The RMA in the context of psychosocial hazard evaluation has several methodological and practical limitations that should be considered. First, our implementation is association-based and relies on continuous self-report outcomes to characterize how psychosocial hazard exposure relates to health-related impairment within a specific organizational context. The outcomes used to operationalize harm should therefore be interpreted within the limits of questionnaire data referring to local characteristics. We do not interpret any of these outcomes as clinical diagnoses; rather, they serve as continuous indicators spanning proximal to more distal health-related impairment as common in occupational risk assessment. Accordingly, the matrix is best understood as a structured prioritization tool for practical application.

Second, although the matrix is displayed separately for each hazard for reasons of transparency and usability, the estimates are derived from multivariable outcome-specific models in which psychosocial hazards are entered simultaneously. This improves the interpretation of hazard-specific associations by accounting for shared variance among hazards, but it also means that individual coefficients may be sensitive to collinearity and suppression effects. Accordingly, the reported coefficients and matrix values should be interpreted as conditional associations within the joint model rather than as isolated effects of single hazards.

Third, our primary specification assumes linear exposure–outcome associations on the COPSOQ 0–100 scale. Hazards are modeled with a linear specification on the 0–100 scale, which constitutes a modeling assumption; future work could examine potential non-linear exposure–outcome relationships. Such extensions may be particularly relevant where theory suggests non-monotonic patterns for certain constructs. In addition, model diagnostics indicated that the linear specification should be understood as an approximation rather than a fully satisfied data-generating model. In particular, heteroscedasticity was observed across the outcome-level models, and there was some evidence of model misspecification, especially for general health. While heteroscedasticity does not necessarily bias the pooled point estimates, it may affect the estimated standard errors and thus the confidence intervals, which are used in the present matrix to communicate uncertainty and support prioritization.

Fourth, as in much organizational research, the study may be affected by common method variance because hazards and outcomes were assessed via self-report questionnaires (Buchanan and Bryman, 2011). The cross-sectional design limits causal inference; therefore, the matrix should be interpreted as describing covariate-adjusted associations rather than causal effects. Moreover, dispositional factors (e.g., negative affectivity, trait resilience) were not measured and may influence both the perception/reporting of psychosocial working conditions and self-rated health, potentially biasing the observed associations. While longitudinal evidence supports health effects of psychosocial working conditions more broadly (Mutambudzi et al., 2019; Andersen et al., 2019; Prakash et al., 2017), future research should validate the proposed matrix framework using longitudinal or quasi-experimental designs and examine its stability across samples and organizational contexts.

Finally, although the current implementation benefits from a large dataset and multiple imputation, smaller organizations or subgroup-level applications may face reduced statistical precision. In such settings, combining the matrix logic with additional data sources (e.g., organizational records, expert assessments) and triangulating findings may be useful (Clarke and Cooper, 2000; Metzler et al., 2019). Overall, our aim is to provide a structured and transparent approach to prioritizing psychosocial hazards in applied settings, while acknowledging the inferential limits of cross-sectional self-report data and model-based translations.

## 5 Conclusion

How to determine at which point a psychosocial hazard poses a risk in need of intervention still challenges scientists and practitioners. Most existing approaches do not sufficiently evaluate hazards, as only few instruments for identifying hazards provide evidence-based threshold values so far. Industrial and organizational psychology has not yet been able to offer sufficient solutions to this problem that unfolds its difficulties especially in practice settings. The risk matrix approach can be regarded as a suitable method for evaluating and prioritizing psychosocial hazards, as it links exposure levels to model-implied changes in health-related outcomes (harm indicators). In our study, we were able to present an empirical investigation of the risk matrix approach for evaluating and prioritizing psychosocial hazards. Our contribution advances evaluation strategies by allowing an empirical assessment of the association between psychosocial hazards and health-related impairment for the respective work situation, regardless of the tool applied for hazard identification. The conceptual considerations and calculations presented can be used as a fundament for further empirical research and, overall, for practice settings. The approach can help organizations and practitioners to establish a risk matrix and thus improving the assessment of psychosocial hazards.

## Data Availability

The datasets presented in this article are not readily available because data that support the findings of this study were generated at a steel manufacturing company. Due to company guidelines data availability is not applicable.
