# Supplementary material for: Managing psychosocial hazards in the workplace: how to link frequency and severity using risk matrices
Source: Front Psychol. 2026 Apr 17;17:1753317. doi: 10.3389/fpsyg.2026.1753317 (PMC13132776; doi:10.3389/fpsyg.2026.1753317)
Supplement: Supplementary file 1 [file Table_1.docx]

Table S1

*Outcome-level model fit and diagnostic summary for the pooled multiple-imputation linear models*

| **Outcome** | **Pooled R² [95% CI]** | **Pooled adjusted R² [95% CI]** | **Mean adjusted R² (range across imputations)** | **Breusch-Pagan tests (p < .05; n/40)** | **RESET tests (p < .05; n/40)** |
| --- | --- | --- | --- | --- | --- |
| Cognitive stress symptoms | 0.293 [0.275, 0.311] | 0.289 [0.271, 0.307] | 0.289 (0.286-0.292) | 40/40 | 19/40 |
| Personal burnout | 0.453 [0.435, 0.470] | 0.450 [0.432, 0.467] | 0.450 (0.444-0.452) | 40/40 | 0/40 |
| General health | 0.105 [0.091, 0.120] | 0.100 [0.086, 0.114] | 0.100 (0.094-0.105) | 40/40 | 40/40 |

*Note.* Values summarize the outcome-level linear models estimated on 40 multiply imputed datasets. Pooled R² and pooled adjusted R² are reported with 95% confidence intervals. Mean adjusted R² is shown together with the observed range across imputations. Breusch-Pagan and RESET columns report the number of imputations (out of 40) with p < .05. These diagnostics were generated from the current outcome-level models including all psychosocial hazards and covariates simultaneously.
