# Supplementary material for: Managing psychosocial hazards in the workplace: how to link frequency and severity using risk matrices
Source: Front Psychol. 2026 Apr 17;17:1753317. doi: 10.3389/fpsyg.2026.1753317 (PMC13132776; doi:10.3389/fpsyg.2026.1753317)
Supplement: Supplementary file 2 [file Table_2.docx]

**Table 1**

Risk-matrix values for quantitative demands

| **Hazard level** | **Cognitive stress symptoms** | **Personal burnout** | **General health** |
| --- | --- | --- | --- |
| 0 | -0.00 [-0.00, 0.00] | -0.00 [-0.00, 0.00] | -0.00 [-0.00, -0.00] |
| 25 | -0.05 [-0.70, 0.60] | -0.52 [-1.26, 0.21] | -2.15 [-3.07, -1.23] |
| 50 | -0.10 [-1.40, 1.19] | -1.05 [-2.52, 0.43] | -4.30 [-6.14, -2.45] |
| 75 | -0.16 [-2.10, 1.79] | -1.57 [-3.78, 0.64] | -6.44 [-9.20, -3.68] |
| 100 | -0.21 [-2.80, 2.38] | -2.09 [-5.04, 0.86] | -8.59 [-12.27, -4.91] |

*Note*. Hazard-specific adjusted matrix values derived from outcome-specific multivariable pooled linear regression models estimated across the multiply imputed datasets (MICE). For each outcome, all psychosocial hazards were entered simultaneously, together with the covariates gender, age, type of work, working hours, work experience, type of contract, and workload. Cells show the predicted change in outcome score (0–100) relative to the reference level (hazard = 0; “never/hardly ever”) at hazard levels 0/25/50/75/100, while holding the remaining hazards and covariates constant; values in brackets are 95% confidence intervals. Higher values indicate worse health for all outcomes.

**Table 2**

Risk-matrix values for emotional demands

| **Hazard level** | **Cognitive stress symptoms** | **Personal burnout** | **General health** |
| --- | --- | --- | --- |
| 0 | 0.00 [0.00, 0.00] | 0.00 [0.00, 0.00] | 0.00 [-0.00, 0.00] |
| 25 | 4.35 [3.80, 4.89] | 2.08 [1.46, 2.70] | 0.40 [-0.39, 1.19] |
| 50 | 8.69 [7.59, 9.79] | 4.16 [2.92, 5.39] | 0.79 [-0.79, 2.37] |
| 75 | 13.04 [11.39, 14.68] | 6.23 [4.38, 8.09] | 1.19 [-1.18, 3.56] |
| 100 | 17.38 [15.19, 19.58] | 8.31 [5.84, 10.78] | 1.58 [-1.58, 4.74] |

*Note*. Hazard-specific adjusted matrix values derived from outcome-specific multivariable pooled linear regression models estimated across the multiply imputed datasets (MICE). For each outcome, all psychosocial hazards were entered simultaneously, together with the covariates gender, age, type of work, working hours, work experience, type of contract, and workload. Cells show the predicted change in outcome score (0–100) relative to the reference level (hazard = 0; “never/hardly ever”) at hazard levels 0/25/50/75/100, while holding the remaining hazards and covariates constant; values in brackets are 95% confidence intervals. Higher values indicate worse health for all outcomes.

**Table 3**

Risk-matrix values for Demands for hiding emotions

| **Hazard level** | **Cognitive stress symptoms** | **Personal burnout** | **General health** |
| --- | --- | --- | --- |
| 0 | 0.00 [-0.00, 0.00] | 0.00 [0.00, 0.00] | 0.00 [0.00, 0.00] |
| 25 | 0.44 [-0.01, 0.89] | 0.72 [0.21, 1.23] | 0.79 [0.14, 1.44] |
| 50 | 0.88 [-0.01, 1.78] | 1.45 [0.42, 2.47] | 1.58 [0.28, 2.89] |
| 75 | 1.33 [-0.02, 2.67] | 2.17 [0.64, 3.70] | 2.37 [0.42, 4.33] |
| 100 | 1.77 [-0.03, 3.56] | 2.89 [0.85, 4.94] | 3.17 [0.55, 5.78] |

*Note*. Hazard-specific adjusted matrix values derived from outcome-specific multivariable pooled linear regression models estimated across the multiply imputed datasets (MICE). For each outcome, all psychosocial hazards were entered simultaneously, together with the covariates gender, age, type of work, working hours, work experience, type of contract, and workload. Cells show the predicted change in outcome score (0–100) relative to the reference level (hazard = 0; “never/hardly ever”) at hazard levels 0/25/50/75/100, while holding the remaining hazards and covariates constant; values in brackets are 95% confidence intervals. Higher values indicate worse health for all outcomes.

**Table 4**

Risk-matrix values for work-privacy conflict

| **Hazard level** | **Cognitive stress symptoms** | **Personal burnout** | **General health** |
| --- | --- | --- | --- |
| 0 | 0.00 [0.00, 0.00] | 0.00 [0.00, 0.00] | 0.00 [0.00, 0.00] |
| 25 | 5.05 [4.62, 5.47] | 3.34 [2.86, 3.83] | 2.15 [1.53, 2.77] |
| 50 | 10.10 [9.24, 10.95] | 6.68 [5.71, 7.66] | 4.30 [3.06, 5.55] |
| 75 | 15.14 [13.87, 16.42] | 10.02 [8.57, 11.48] | 6.45 [4.58, 8.32] |
| 100 | 20.19 [18.49, 21.90] | 13.37 [11.42, 15.31] | 8.60 [6.11, 11.09] |

*Note*. Hazard-specific adjusted matrix values derived from outcome-specific multivariable pooled linear regression models estimated across the multiply imputed datasets (MICE). For each outcome, all psychosocial hazards were entered simultaneously, together with the covariates gender, age, type of work, working hours, work experience, type of contract, and workload. Cells show the predicted change in outcome score (0–100) relative to the reference level (hazard = 0; “never/hardly ever”) at hazard levels 0/25/50/75/100, while holding the remaining hazards and covariates constant; values in brackets are 95% confidence intervals. Higher values indicate worse health for all outcomes.

**Table 5**

Risk-matrix values for influence at work

| **Hazard level** | **Cognitive stress symptoms** | **Personal burnout** | **General health** |
| --- | --- | --- | --- |
| 0 | 0.00 [0.00, 0.00] | 0.00 [0.00, 0.00] | -0.00 [-0.00, 0.00] |
| 25 | 1.08 [0.59, 1.57] | 0.57 [0.00, 1.13] | -0.19 [-0.90, 0.51] |
| 50 | 2.15 [1.17, 3.13] | 1.13 [0.00, 2.26] | -0.39 [-1.79, 1.01] |
| 75 | 3.23 [1.76, 4.70] | 1.70 [0.00, 3.39] | -0.58 [-2.69, 1.52] |
| 100 | 4.30 [2.34, 6.26] | 2.26 [0.00, 4.52] | -0.78 [-3.58, 2.02] |

*Note*. Hazard-specific adjusted matrix values derived from outcome-specific multivariable pooled linear regression models estimated across the multiply imputed datasets (MICE). For each outcome, all psychosocial hazards were entered simultaneously, together with the covariates gender, age, type of work, working hours, work experience, type of contract, and workload. Cells show the predicted change in outcome score (0–100) relative to the reference level (hazard = 0; “never/hardly ever”) at hazard levels 0/25/50/75/100, while holding the remaining hazards and covariates constant; values in brackets are 95% confidence intervals. Higher values indicate worse health for all outcomes.

**Table 6**

Risk-matrix values for degrees of freedom at work

| **Hazard level** | **Cognitive stress symptoms** | **Personal burnout** | **General health** |
| --- | --- | --- | --- |
| 0 | -0.00 [-0.00, 0.00] | -0.00 [-0.00, -0.00] | 0.00 [0.00, 0.00] |
| 25 | -0.27 [-0.84, 0.30] | -1.08 [-1.73, -0.43] | 0.96 [0.12, 1.80] |
| 50 | -0.54 [-1.68, 0.60] | -2.16 [-3.45, -0.87] | 1.93 [0.25, 3.61] |
| 75 | -0.81 [-2.52, 0.91] | -3.24 [-5.18, -1.30] | 2.89 [0.37, 5.41] |
| 100 | -1.07 [-3.36, 1.21] | -4.32 [-6.91, -1.73] | 3.86 [0.50, 7.22] |

*Note*. Hazard-specific adjusted matrix values derived from outcome-specific multivariable pooled linear regression models estimated across the multiply imputed datasets (MICE). For each outcome, all psychosocial hazards were entered simultaneously, together with the covariates gender, age, type of work, working hours, work experience, type of contract, and workload. Cells show the predicted change in outcome score (0–100) relative to the reference level (hazard = 0; “never/hardly ever”) at hazard levels 0/25/50/75/100, while holding the remaining hazards and covariates constant; values in brackets are 95% confidence intervals. Higher values indicate worse health for all outcomes.

**Table 7**

Risk-matrix values for possibilities for development

| **Hazard level** | **Cognitive stress symptoms** | **Personal burnout** | **General health** |
| --- | --- | --- | --- |
| 0 | 0.00 [0.00, 0.00] | 0.00 [-0.00, 0.00] | -0.00 [-0.00, 0.00] |
| 25 | 1.38 [0.77, 1.98] | 0.51 [-0.18, 1.19] | -0.10 [-0.99, 0.79] |
| 50 | 2.75 [1.55, 3.96] | 1.01 [-0.36, 2.39] | -0.20 [-1.97, 1.57] |
| 75 | 4.13 [2.32, 5.93] | 1.52 [-0.54, 3.58] | -0.30 [-2.96, 2.36] |
| 100 | 5.51 [3.10, 7.91] | 2.03 [-0.72, 4.77] | -0.40 [-3.94, 3.14] |

*Note*. Hazard-specific adjusted matrix values derived from outcome-specific multivariable pooled linear regression models estimated across the multiply imputed datasets (MICE). For each outcome, all psychosocial hazards were entered simultaneously, together with the covariates gender, age, type of work, working hours, work experience, type of contract, and workload. Cells show the predicted change in outcome score (0–100) relative to the reference level (hazard = 0; “never/hardly ever”) at hazard levels 0/25/50/75/100, while holding the remaining hazards and covariates constant; values in brackets are 95% confidence intervals. Higher values indicate worse health for all outcomes.

**Table 8**

Risk-matrix values for meaning of work

| **Hazard level** | **Cognitive stress symptoms** | **Personal burnout** | **General health** |
| --- | --- | --- | --- |
| 0 | 0.00 [0.00, 0.00] | 0.00 [0.00, 0.00] | 0.00 [-0.00, 0.00] |
| 25 | 1.29 [0.66, 1.91] | 1.27 [0.55, 1.99] | 0.01 [-0.90, 0.91] |
| 50 | 2.57 [1.32, 3.82] | 2.54 [1.10, 3.98] | 0.01 [-1.80, 1.83] |
| 75 | 3.86 [1.99, 5.73] | 3.81 [1.65, 5.97] | 0.02 [-2.71, 2.74] |
| 100 | 5.14 [2.65, 7.64] | 5.08 [2.20, 7.96] | 0.02 [-3.61, 3.66] |

*Note*. Hazard-specific adjusted matrix values derived from outcome-specific multivariable pooled linear regression models estimated across the multiply imputed datasets (MICE). For each outcome, all psychosocial hazards were entered simultaneously, together with the covariates gender, age, type of work, working hours, work experience, type of contract, and workload. Cells show the predicted change in outcome score (0–100) relative to the reference level (hazard = 0; “never/hardly ever”) at hazard levels 0/25/50/75/100, while holding the remaining hazards and covariates constant; values in brackets are 95% confidence intervals. Higher values indicate worse health for all outcomes.

**Table 9**

Risk-matrix values for commitment to the workplace

| **Hazard level** | **Cognitive stress symptoms** | **Personal burnout** | **General health** |
| --- | --- | --- | --- |
| 0 | 0.00 [-0.00, 0.00] | -0.00 [-0.00, 0.00] | 0.00 [0.00, 0.00] |
| 25 | 0.55 [-0.03, 1.12] | -0.52 [-1.17, 0.13] | 1.13 [0.32, 1.94] |
| 50 | 1.09 [-0.05, 2.24] | -1.04 [-2.34, 0.26] | 2.26 [0.64, 3.88] |
| 75 | 1.64 [-0.08, 3.36] | -1.56 [-3.51, 0.39] | 3.39 [0.96, 5.82] |
| 100 | 2.19 [-0.10, 4.48] | -2.08 [-4.68, 0.52] | 4.52 [1.28, 7.76] |

*Note*. Hazard-specific adjusted matrix values derived from outcome-specific multivariable pooled linear regression models estimated across the multiply imputed datasets (MICE). For each outcome, all psychosocial hazards were entered simultaneously, together with the covariates gender, age, type of work, working hours, work experience, type of contract, and workload. Cells show the predicted change in outcome score (0–100) relative to the reference level (hazard = 0; “never/hardly ever”) at hazard levels 0/25/50/75/100, while holding the remaining hazards and covariates constant; values in brackets are 95% confidence intervals. Higher values indicate worse health for all outcomes.

**Table 10**

Risk-matrix values for predictability

| **Hazard level** | **Cognitive stress symptoms** | **Personal burnout** | **General health** |
| --- | --- | --- | --- |
| 0 | -0.00 [-0.00, 0.00] | -0.00 [-0.00, -0.00] | -0.00 [-0.00, -0.00] |
| 25 | -0.52 [-1.05, 0.01] | -0.90 [-1.51, -0.29] | -0.90 [-1.65, -0.14] |
| 50 | -1.04 [-2.10, 0.02] | -1.81 [-3.03, -0.59] | -1.79 [-3.29, -0.29] |
| 75 | -1.56 [-3.15, 0.03] | -2.71 [-4.54, -0.88] | -2.69 [-4.94, -0.43] |
| 100 | -2.08 [-4.20, 0.04] | -3.61 [-6.05, -1.17] | -3.58 [-6.59, -0.57] |

*Note*. Hazard-specific adjusted matrix values derived from outcome-specific multivariable pooled linear regression models estimated across the multiply imputed datasets (MICE). For each outcome, all psychosocial hazards were entered simultaneously, together with the covariates gender, age, type of work, working hours, work experience, type of contract, and workload. Cells show the predicted change in outcome score (0–100) relative to the reference level (hazard = 0; “never/hardly ever”) at hazard levels 0/25/50/75/100, while holding the remaining hazards and covariates constant; values in brackets are 95% confidence intervals. Higher values indicate worse health for all outcomes.

**Table 11**

Risk-matrix values for role clarity

| **Hazard level** | **Cognitive stress symptoms** | **Personal burnout** | **General health** |
| --- | --- | --- | --- |
| 0 | 0.00 [0.00, 0.00] | 0.00 [0.00, 0.00] | 0.00 [0.00, 0.00] |
| 25 | 1.30 [0.66, 1.94] | 4.34 [3.61, 5.07] | 1.47 [0.56, 2.38] |
| 50 | 2.61 [1.33, 3.88] | 8.68 [7.22, 10.14] | 2.94 [1.12, 4.76] |
| 75 | 3.91 [1.99, 5.82] | 13.02 [10.84, 15.20] | 4.42 [1.69, 7.14] |
| 100 | 5.21 [2.66, 7.76] | 17.36 [14.45, 20.27] | 5.89 [2.25, 9.52] |

*Note*. Hazard-specific adjusted matrix values derived from outcome-specific multivariable pooled linear regression models estimated across the multiply imputed datasets (MICE). For each outcome, all psychosocial hazards were entered simultaneously, together with the covariates gender, age, type of work, working hours, work experience, type of contract, and workload. Cells show the predicted change in outcome score (0–100) relative to the reference level (hazard = 0; “never/hardly ever”) at hazard levels 0/25/50/75/100, while holding the remaining hazards and covariates constant; values in brackets are 95% confidence intervals. Higher values indicate worse health for all outcomes.

**Table 12**

Risk-matrix values for role conflicts

| **Hazard level** | **Cognitive stress symptoms** | **Personal burnout** | **General health** |
| --- | --- | --- | --- |
| 0 | 0.00 [0.00, 0.00] | 0.00 [0.00, 0.00] | 0.00 [-0.00, 0.00] |
| 25 | 1.49 [0.96, 2.03] | 1.58 [0.96, 2.19] | 0.62 [-0.13, 1.38] |
| 50 | 2.99 [1.91, 4.06] | 3.15 [1.93, 4.38] | 1.25 [-0.27, 2.77] |
| 75 | 4.48 [2.87, 6.10] | 4.73 [2.89, 6.57] | 1.87 [-0.40, 4.15] |
| 100 | 5.98 [3.82, 8.13] | 6.31 [3.86, 8.76] | 2.50 [-0.54, 5.53] |

*Note*. Hazard-specific adjusted matrix values derived from outcome-specific multivariable pooled linear regression models estimated across the multiply imputed datasets (MICE). For each outcome, all psychosocial hazards were entered simultaneously, together with the covariates gender, age, type of work, working hours, work experience, type of contract, and workload. Cells show the predicted change in outcome score (0–100) relative to the reference level (hazard = 0; “never/hardly ever”) at hazard levels 0/25/50/75/100, while holding the remaining hazards and covariates constant; values in brackets are 95% confidence intervals. Higher values indicate worse health for all outcomes.

**Table 13**

Risk-matrix values for quality of leadership

| **Hazard level** | **Cognitive stress symptoms** | **Personal burnout** | **General health** |
| --- | --- | --- | --- |
| 0 | 0.00 [-0.00, 0.00] | -0.00 [-0.00, 0.00] | 0.00 [0.00, 0.00] |
| 25 | 0.35 [-0.19, 0.89] | -0.27 [-0.89, 0.36] | 1.16 [0.37, 1.94] |
| 50 | 0.71 [-0.38, 1.79] | -0.54 [-1.79, 0.71] | 2.31 [0.75, 3.88] |
| 75 | 1.06 [-0.56, 2.68] | -0.80 [-2.68, 1.07] | 3.47 [1.12, 5.83] |
| 100 | 1.41 [-0.75, 3.57] | -1.07 [-3.57, 1.42] | 4.63 [1.49, 7.77] |

*Note*. Hazard-specific adjusted matrix values derived from outcome-specific multivariable pooled linear regression models estimated across the multiply imputed datasets (MICE). For each outcome, all psychosocial hazards were entered simultaneously, together with the covariates gender, age, type of work, working hours, work experience, type of contract, and workload. Cells show the predicted change in outcome score (0–100) relative to the reference level (hazard = 0; “never/hardly ever”) at hazard levels 0/25/50/75/100, while holding the remaining hazards and covariates constant; values in brackets are 95% confidence intervals. Higher values indicate worse health for all outcomes.

**Table 14**

Risk-matrix values for social support

| **Hazard level** | **Cognitive stress symptoms** | **Personal burnout** | **General health** |
| --- | --- | --- | --- |
| 0 | 0.00 [-0.00, 0.00] | 0.00 [-0.00, 0.00] | 0.00 [-0.00, 0.00] |
| 25 | 0.02 [-0.65, 0.68] | 0.01 [-0.75, 0.77] | 0.50 [-0.46, 1.46] |
| 50 | 0.03 [-1.29, 1.36] | 0.02 [-1.50, 1.55] | 1.00 [-0.91, 2.92] |
| 75 | 0.05 [-1.94, 2.03] | 0.03 [-2.26, 2.32] | 1.51 [-1.37, 4.38] |
| 100 | 0.06 [-2.59, 2.71] | 0.04 [-3.01, 3.10] | 2.01 [-1.82, 5.84] |

*Note*. Hazard-specific adjusted matrix values derived from outcome-specific multivariable pooled linear regression models estimated across the multiply imputed datasets (MICE). For each outcome, all psychosocial hazards were entered simultaneously, together with the covariates gender, age, type of work, working hours, work experience, type of contract, and workload. Cells show the predicted change in outcome score (0–100) relative to the reference level (hazard = 0; “never/hardly ever”) at hazard levels 0/25/50/75/100, while holding the remaining hazards and covariates constant; values in brackets are 95% confidence intervals. Higher values indicate worse health for all outcomes.

**Table 15**

Risk-matrix values for feedback

| **Hazard level** | **Cognitive stress symptoms** | **Personal burnout** | **General health** |
| --- | --- | --- | --- |
| 0 | -0.00 [-0.00, 0.00] | -0.00 [-0.00, 0.00] | -0.00 [-0.00, 0.00] |
| 25 | -0.19 [-0.67, 0.29] | -0.35 [-0.91, 0.20] | -0.27 [-0.98, 0.45] |
| 50 | -0.38 [-1.34, 0.58] | -0.71 [-1.82, 0.41] | -0.53 [-1.96, 0.89] |
| 75 | -0.57 [-2.01, 0.88] | -1.06 [-2.73, 0.61] | -0.80 [-2.94, 1.34] |
| 100 | -0.76 [-2.68, 1.17] | -1.41 [-3.64, 0.82] | -1.07 [-3.92, 1.79] |

*Note*. Hazard-specific adjusted matrix values derived from outcome-specific multivariable pooled linear regression models estimated across the multiply imputed datasets (MICE). For each outcome, all psychosocial hazards were entered simultaneously, together with the covariates gender, age, type of work, working hours, work experience, type of contract, and workload. Cells show the predicted change in outcome score (0–100) relative to the reference level (hazard = 0; “never/hardly ever”) at hazard levels 0/25/50/75/100, while holding the remaining hazards and covariates constant; values in brackets are 95% confidence intervals. Higher values indicate worse health for all outcomes.

**Table 16**

Risk-matrix values for social relations

| **Hazard level** | **Cognitive stress symptoms** | **Personal burnout** | **General health** |
| --- | --- | --- | --- |
| 0 | -0.00 [-0.00, 0.00] | -0.00 [-0.00, 0.00] | -0.00 [-0.00, 0.00] |
| 25 | -0.24 [-0.66, 0.17] | -0.21 [-0.68, 0.26] | -0.25 [-0.84, 0.34] |
| 50 | -0.49 [-1.32, 0.34] | -0.41 [-1.36, 0.53] | -0.50 [-1.69, 0.68] |
| 75 | -0.73 [-1.98, 0.51] | -0.62 [-2.03, 0.79] | -0.75 [-2.53, 1.02] |
| 100 | -0.97 [-2.63, 0.68] | -0.83 [-2.71, 1.06] | -1.01 [-3.37, 1.36] |

*Note*. Hazard-specific adjusted matrix values derived from outcome-specific multivariable pooled linear regression models estimated across the multiply imputed datasets (MICE). For each outcome, all psychosocial hazards were entered simultaneously, together with the covariates gender, age, type of work, working hours, work experience, type of contract, and workload. Cells show the predicted change in outcome score (0–100) relative to the reference level (hazard = 0; “never/hardly ever”) at hazard levels 0/25/50/75/100, while holding the remaining hazards and covariates constant; values in brackets are 95% confidence intervals. Higher values indicate worse health for all outcomes.

**Table 17**

Risk-matrix values for sense of community

| **Hazard level** | **Cognitive stress symptoms** | **Personal burnout** | **General health** |
| --- | --- | --- | --- |
| 0 | 0.00 [0.00, 0.00] | 0.00 [0.00, 0.00] | 0.00 [0.00, 0.00] |
| 25 | 1.53 [0.95, 2.11] | 2.58 [1.91, 3.25] | 1.89 [1.05, 2.74] |
| 50 | 3.06 [1.90, 4.23] | 5.17 [3.83, 6.51] | 3.79 [2.10, 5.48] |
| 75 | 4.60 [2.85, 6.34] | 7.75 [5.74, 9.76] | 5.68 [3.14, 8.21] |
| 100 | 6.13 [3.80, 8.46] | 10.34 [7.65, 13.02] | 7.57 [4.19, 10.95] |

*Note*. Hazard-specific adjusted matrix values derived from outcome-specific multivariable pooled linear regression models estimated across the multiply imputed datasets (MICE). For each outcome, all psychosocial hazards were entered simultaneously, together with the covariates gender, age, type of work, working hours, work experience, type of contract, and workload. Cells show the predicted change in outcome score (0–100) relative to the reference level (hazard = 0; “never/hardly ever”) at hazard levels 0/25/50/75/100, while holding the remaining hazards and covariates constant; values in brackets are 95% confidence intervals. Higher values indicate worse health for all outcomes.

**Table 18**

Risk-matrix values for signs of bullying

| **Hazard level** | **Cognitive stress symptoms** | **Personal burnout** | **General health** |
| --- | --- | --- | --- |
| 0 | 0.00 [0.00, 0.00] | 0.00 [0.00, 0.00] | 0.00 [0.00, 0.00] |
| 25 | 1.28 [0.89, 1.68] | 1.78 [1.33, 2.22] | 1.03 [0.45, 1.62] |
| 50 | 2.56 [1.77, 3.35] | 3.55 [2.67, 4.44] | 2.07 [0.89, 3.24] |
| 75 | 3.84 [2.66, 5.03] | 5.33 [4.00, 6.66] | 3.10 [1.34, 4.86] |
| 100 | 5.13 [3.55, 6.70] | 7.11 [5.34, 8.88] | 4.14 [1.79, 6.49] |

*Note*. Hazard-specific adjusted matrix values derived from outcome-specific multivariable pooled linear regression models estimated across the multiply imputed datasets (MICE). For each outcome, all psychosocial hazards were entered simultaneously, together with the covariates gender, age, type of work, working hours, work experience, type of contract, and workload. Cells show the predicted change in outcome score (0–100) relative to the reference level (hazard = 0; “never/hardly ever”) at hazard levels 0/25/50/75/100, while holding the remaining hazards and covariates constant; values in brackets are 95% confidence intervals. Higher values indicate worse health for all outcomes.

**Table 19**

Risk-matrix values for trust and justice

| **Hazard level** | **Cognitive stress symptoms** | **Personal burnout** | **General health** |
| --- | --- | --- | --- |
| 0 | 0.00 [0.00, 0.00] | 0.00 [-0.00, 0.00] | -0.00 [-0.00, 0.00] |
| 25 | 0.89 [0.22, 1.56] | 0.47 [-0.29, 1.23] | -0.08 [-1.06, 0.90] |
| 50 | 1.78 [0.44, 3.11] | 0.94 [-0.57, 2.46] | -0.16 [-2.12, 1.80] |
| 75 | 2.67 [0.67, 4.67] | 1.42 [-0.86, 3.70] | -0.24 [-3.18, 2.69] |
| 100 | 3.56 [0.89, 6.23] | 1.89 [-1.15, 4.93] | -0.32 [-4.24, 3.59] |

*Note*. Hazard-specific adjusted matrix values derived from outcome-specific multivariable pooled linear regression models estimated across the multiply imputed datasets (MICE). For each outcome, all psychosocial hazards were entered simultaneously, together with the covariates gender, age, type of work, working hours, work experience, type of contract, and workload. Cells show the predicted change in outcome score (0–100) relative to the reference level (hazard = 0; “never/hardly ever”) at hazard levels 0/25/50/75/100, while holding the remaining hazards and covariates constant; values in brackets are 95% confidence intervals. Higher values indicate worse health for all outcomes.

**Table 20**

Risk-matrix values for job insecurity

| **Hazard level** | **Cognitive stress symptoms** | **Personal burnout** | **General health** |
| --- | --- | --- | --- |
| 0 | 0.00 [0.00, 0.00] | 0.00 [0.00, 0.00] | 0.00 [-0.00, 0.00] |
| 25 | 2.55 [2.17, 2.93] | 2.17 [1.74, 2.61] | 0.01 [-0.54, 0.55] |
| 50 | 5.10 [4.35, 5.86] | 4.35 [3.48, 5.21] | 0.01 [-1.08, 1.11] |
| 75 | 7.65 [6.52, 8.79] | 6.52 [5.22, 7.82] | 0.02 [-1.62, 1.66] |
| 100 | 10.21 [8.69, 11.72] | 8.69 [6.96, 10.43] | 0.03 [-2.16, 2.22] |

*Note*. Hazard-specific adjusted matrix values derived from outcome-specific multivariable pooled linear regression models estimated across the multiply imputed datasets (MICE). For each outcome, all psychosocial hazards were entered simultaneously, together with the covariates gender, age, type of work, working hours, work experience, type of contract, and workload. Cells show the predicted change in outcome score (0–100) relative to the reference level (hazard = 0; “never/hardly ever”) at hazard levels 0/25/50/75/100, while holding the remaining hazards and covariates constant; values in brackets are 95% confidence intervals. Higher values indicate worse health for all outcomes.

**Table 21**

Risk-matrix values for work environment

| **Hazard level** | **Cognitive stress symptoms** | **Personal burnout** | **General health** |
| --- | --- | --- | --- |
| 0 | 0.00 [0.00, 0.00] | 0.00 [0.00, 0.00] | 0.00 [0.00, 0.00] |
| 25 | 4.49 [3.88, 5.10] | 3.88 [3.18, 4.58] | 1.03 [0.16, 1.90] |
| 50 | 8.98 [7.76, 10.20] | 7.76 [6.35, 9.16] | 2.06 [0.33, 3.79] |
| 75 | 13.46 [11.63, 15.30] | 11.64 [9.53, 13.74] | 3.09 [0.49, 5.69] |
| 100 | 17.95 [15.51, 20.39] | 15.51 [12.70, 18.33] | 4.12 [0.65, 7.58] |

*Note*. Hazard-specific adjusted matrix values derived from outcome-specific multivariable pooled linear regression models estimated across the multiply imputed datasets (MICE). For each outcome, all psychosocial hazards were entered simultaneously, together with the covariates gender, age, type of work, working hours, work experience, type of contract, and workload. Cells show the predicted change in outcome score (0–100) relative to the reference level (hazard = 0; “never/hardly ever”) at hazard levels 0/25/50/75/100, while holding the remaining hazards and covariates constant; values in brackets are 95% confidence intervals. Higher values indicate worse health for all outcomes.
